# Machine learning techniques for predicting depression and anxiety in pregnant and postpartum women during the COVID-19 pandemic: a cross-sectional regional study

**DOI:** 10.12688/f1000research.110090.1

**Published:** 2022-04-04

**Authors:** Radwan Qasrawi, Malak Amro, Stephanny VicunaPolo, Diala Abu Al-Halawa, Hazem Agha, Rania Abu Seir, Maha Hoteit, Reem Hoteit, Sabika Allehdan, Nouf Behzad, Khlood Bookari, Majid AlKhalaf, Haleama Al-Sabbah, Eman Badran, Reema Tayyem

**Affiliations:** 1 Department of Computer Science, Al- Quds University, Jerusalem, Palestinian Territory; 2 Dpertment of Computer Engineering, Istinye University, Istanbul, 34010, Turkey; 3 Department of Faculty of Medicine, Al- Quds University, Jerusalem, Palestinian Territory; 4 Department of Medical Laboratory Sciences, Al-Quds University, Jerusalem, Palestinian Territory; 5 Faculty of Public Health, Lebanese University, Beirut, Lebanon; 6 PHENOL Research Group (Public Health Nutrition Program Lebanon), Faculty of Public Health, Lebanese University, Beirut, Lebanon; 7 Lebanese University Nutrition Surveillance Center (LUNSC), Lebanese Food Drugs and Chemical Administrations, Lebanese University, Beirut, Lebanon; 8 Clinical Research Institute, American University of Beirut, Bliss Street, Riad El Solh 1107 2020, Beirut, Lebanon; 9 Department of Biology, College of Science, University of Bahrain, Zallaq, Bahrain; 10 Salmaniya Medical Complex, Ministry of Health, Manama, Bahrain; 11 Department of Clinical Nutrition, Faculty of Applied Medical Sciences, Taibah University, Medna, Saudi Arabia; 12 National Nutrition Committee (NNC), Saudi Food and Drug Authority (Saudi FDA), Riyadh, Saudi Arabia; 13 Department of Health Sciences, Zayed University, Dubai, United Arab Emirates; 14 Faculty of Medicine, University of Jordan, Amman, Jordan; 15 Department of Human Nutrition, College of Health Sciences, Qatar University, Doha, Qatar; 16 Department of Nutrition and Food Technology, Faculty of Agriculture, The University of Jordan, Amman, 11942, Jordan

**Keywords:** Machine Learning, Anxiety, Depression, Pregnancy, COVID-19, Random Forest

## Abstract

**Background**: Maternal depression and anxiety are significant public health concerns that play an important role in the health and well-being of mothers and children. The COVID-19 pandemic, the consequential lockdowns and related safety restrictions worldwide negatively affected the mental health of pregnant and postpartum women.

**Methods:** This regional study aimed to develop a machine learning (ML) model for the prediction of maternal depression and anxiety. The study used a dataset collected from five Arab countries during the COVID-19 pandemic between July to December 2020. The population sample included 3569 women (1939 pregnant and 1630 postpartum) from five countries (Jordan, Palestine, Lebanon, Saudi Arabia, and Bahrain). The performance of seven machine learning algorithms was assessed for the prediction of depression and anxiety symptoms.

**Results**: The Gradient Boosting (GB) and Random Forest (RF) models outperformed other studied ML algorithms with accuracy values of 83.3% and 83.2% for depression, respectively, and values of 82.9% and 81.3% for anxiety, respectively. The Mathew’s Correlation Coefficient was evaluated for the ML models; the Naïve Bayes (NB) and GB models presented the highest performance measures (0.63 and 0.59) for depression and (0.74 and 0.73) for anxiety, respectively. The features’ importance ranking was evaluated, the results showed that stress during pregnancy, family support, financial issues, income, and social support were the most significant values in predicting anxiety and depression.

**Conclusion:** Overall, the study evidenced the power of ML models in predicting maternal depression and anxiety and proved to be an efficient tool for identifying and predicting the associated risk factors that influence maternal mental health. The deployment of machine learning models for screening and early detection of depression and anxiety among pregnant and postpartum women might facilitate the development of health prevention and intervention programs that will enhance maternal and child health in low- and middle-income countries.

## Introduction

The emergence of the Coronavirus disease (COVID-19) in late 2019 and early 2020 has severely impacted the global population. Being characterized as an infectious disease primarily spreading through droplets of saliva or nasal discharge ^1^, the infection rate is significant and its consequences can be lethal ^2– 4^. The disease is particularly dangerous to vulnerable populations, such as the elderly and those with underlying medical conditions including cardiovascular disease, diabetes, respiratory disease, and cancer ^3– 5^. Nonetheless, the specific implications of COVID-19 infection on pregnancy and childbirth have remained unidentified throughout the pandemic ^6– 9^.

The uncertainty about the nature, transmission, and mortality of the virus, together with its rapid spread and the consequential social and mobility restrictions (quarantines, lockdowns, and social distancing) have impacted the mental health of pregnant women worldwide ^5, 10^. In fact, the psychological effects of COVID-19 on pregnant women may lead to the appearance or increment of stress, anxiety, and depression symptoms as indicated in Broche-Perez *et al*. study ^11^. In a 2020 study by Tokgoz *et al*. ^12^, the authors demonstrated that pregnant women during the COVID-19 pandemic presented higher rates of depression, stress, and anxiety than pregnant women before the pandemic. The study further evidenced that mental health disorders during pregnancy can result in pre-term labour, low birth weight, delayed neuropsychiatric development in children, preeclampsia, and unscheduled caesarean delivery ^11, 13^.

However, mental health disorders among pregnant women are widely undiagnosed and could result in worse consequences for mother and child ^14, 15^. Furthermore, the traditionally applied screening programs for psychological conditions rely on self-reporting and are for the most part designed to detect the population with pre-existing symptoms ^6, 16^. Contrary to the available traditional assessment of psychological disorders, artificial intelligence (AI) models can predict potential incidence of depression and anxiety among pregnant women, which would facilitate pre-emptive action, treatment, and early diagnosis ^16, 17^. As a matter of fact, one subarea of AI, machine learning (ML), has previously been used in the field of mental health for the prediction of psychological conditions such as anxiety, depression, obsessive-compulsive disorder (OCD), and post-traumatic stress disorder (PTSD), both prior to and during the onset of the COVID-19 pandemic ^18, 19^.

In a 2020 study by Seah *et al*. ^20^, five ML algorithms where applied for the prediction of anxiety, depression, and stress on individuals around the world using the Depression, Anxiety and Stress Scale questionnaire ( DASS 21). The Random Forest classifier, a machine learning algorithm used for data classification, had the best performance accuracy in predicting psychological conditions. A study by Priya *et al*. ^21^, utilized ML tools for the creation of a new diagnostic methodology for anxiety and depression to replace traditional diagnosis through self-reported symptoms. The tool represented an improvement in diagnosis and treatment. Similarly, a study by Richter *et al*. ^22^ applied eight ML algorithms, including a hybrid model, for the prediction of psychological problems such as anxiety, depression, and stress. The study found that the hybrid model presented higher accuracy rates than the single algorithms used. In relation to maternal health, only a few studies have been found to use ML models for the prediction of psychological disorders ^16, 19, 23, 24^. Among the available studies in this field, a study by Shin *et al*. ^24^, developed a predictive model for postpartum depression using nine different ML approaches, the results showed that the Random Forest model achieved the highest accuracy rates. In addition, the study of Hochman *et al.* ^16^, provided evidence that ML models are able to accurately screen and identify populations at high risk of postpartum depression for preventive intervention.

Thus, the use of ML techniques in mental health prediction and diagnosis might yield positive results in the reduction of self-harm and the provision of timely treatment for at-risk patients. However, very limited studies have used ML in maternal mental health, especially in relation to COVID-19 ^11, 16, 19, 24^. This study aims to enrich the literature by assessing the performance of ML techniques in studying the effect of the COVID-19 lockdown on maternal mental health in low- and middle-income countries. The study used ML for predicting depression and anxiety symptoms from different features during the COVID-19 lockdown. To date, this is the first international study using population-based datasets from five countries (Palestine, Lebanon, Jordan, Saudi Arabia, and Bahrain) that accounts for multiple maternal and mental health variables. 

## Methods

### Ethics

The study obtained written approval of the Ethics Committee in Scientific Research of University of Jordan, Jordan (19/2020/585), as well as universities from all participating countries. Written informed consent was obtained from all participants.

***Data set*.** This study is the first of its kind as it utilized a regional dataset for evaluating the performance of ML algorithms in predicting depression and anxiety among pregnant and postpartum women during the COVID-19 lockdown in five Arab countries. The stratification of participants into different sets of data according to country of residence and the overall prediction for the total participants provide important and interesting information about the effect of the COVID-19 lockdown on pregnant women. A total of 3,569 women (1,939 pregnant and 1,630 postpartum) from five countries (Jordan, Palestine, Lebanon, Saudi Arabia, and Bahrain) participated in the study. Data were collected during the period of lockdown from July to December 2020.

The data set was extracted from a regional study conducted by the authors for assessing the impact of the COVID-19 pandemic on pregnant and postpartum women's physical and mental health. The study collected data from five Arab countries including: Lebanon, Palestine, Jordan, Bahrain, and Saudi Arabia. A total of 3569 women (currently pregnant or were pregnant during the COVID-19 pandemic lockdown) were selected in this study. The data set including socio-demographic variables and risk factors related to depression, anxiety, and physical and mental health among pregnant women is shown in Table 1.

**Table 1.** Machine learning models variables descriptions.

Variable	Description	Value
**Country**	Country of residence	Jordan; Palestine; Lebanon; Saudi Arabia; Bahrain
**Locality**	Place of residence	Urban, non-urban
**Age**	Women age	0)≤20;1)21-29; 2)30-39; 4)40+
**Marriage age**	Women’s age at marriage	0)≤18;1)19-25; 2)26-30; 3)31+
**W_education**	Women’s education level	0)≤ Secondary; 1) Diploma; 2) Bachelor +
**Preg_freq**	Number of pregnancies	One; Two; Three +
**Abortion_freq**	Number of miscarriages	Zero; One; Two +
**Income**	Family Income during COVID-19	1)Decreased; 2) Increased; 3) Remain the same
**W_work**	Women’s working status	Yes, No
**Pha**	Physical activity level	Inactive (<1/2 hour); Active (>=1/2 hour)
**Ta**	Technology activities	Yes, No
**Healthy intake**	Healthy food consumption	Yes, No
**Unhealthy intake**	Unhealthy food consumption	Yes, No
**Diagnosed_C19**	COVID-19 diagnosis	Yes, No
**Relatives_diagnosed**	One of your relatives diagnosed with COVID-19	Yes, No
**Health_prob**	Has any health problem	Yes, No
**Cancelling app**	Cancelling clinic visit due to lockdown	Yes; No
**Smoking**	Are you smoking?	Yes; No
**Sleeping**	Sleeping hours during pandemic (hours per day)	0) <6; 1) 6-8; 2) >8 hours/day
**Preg_depression**	Depression level	Low; moderate; high
**Preg_anxiety**	Anxiety level	Low; moderate; high
**Preg_stress**	Stress level	Low; moderate; high

### Questionnaire

A cross-sectional study design was used for collecting the study data during the COVID-19 pandemic from August to November 2020, in the five listed countries: Lebanon, Palestine, Jordan, Bahrain, and Saudi Arabia) in the Arab region. The snowball sampling method was used to recruit pregnant women. The initial participants were contacted through the research team’s professional network, and the obstetric and maternity clinics in the participating countries. Data was collected through a web-based questionnaire, which was previously validated in two published studies ^25, 26^, and the software was designed by Palestinian National Nutrition Platform. The questionnaire was disseminated by researchers through their social media networks ( Facebook, WhatsApp, and Instagram), and the participating universities network. Furthermore, hard copies of the questionnaire were distributed to women in some areas with limited internet access through obstetric and maternity clinics. The survey considered several sociodemographic features of pregnant women, medical history, nutrition patterns, physical activity, smoking, education, residency, economic situation, anxiety indicators, and depression indicators. Questions regarding the pre-pregnancy period were not included in the survey. For the full questionnaire, see *Extended data* ^27^.

### Criteria

The following criteria guided the data collection process: (i) pregnancy during the COVID-19 pandemic period; (ii) normal pregnancy (i.e., no complications); (iii) aged over 18 (iv) place of residence (the five study countries); (v) having answered all questions in the questionnaire. Moreover, the exclusion criteria included conception during the intra-COVID19 pandemic period, as well as risk factors such as miscarriage and chronic health complications.

***Outcome variables*.** The outcome variables included pregnant women's depression and anxiety levels. Participants were assessed for depression and anxiety using the Patient Health Questionnaire ( PHQ-9) and Generalized Anxiety Disorder ( GAD-7) scales.

*Depression:* The depression data was collected using the Patient Health Questionnaire (PHQ), a self-reported scale designed by 28 to screen for symptoms of depression. The PHQ items are composed of four answer categories (Never =0; several days =1, more than half of the days =2, and nearly every day =3). The total score was calculated by summing the scale items responses. The PHQ total score was classified into the following groups: Low=0; Moderate=1; High =2.

*Anxiety:* The GAD-7 scale ^28^ was used for measuring generalized anxiety disorder. The anxiety score was estimated by assigning scores of 0, 1, 2, and 3 to the response categories of (Never =0; several days =1, more than half of the days =2, and nearly every day =3). The total score was calculated by summing the scale items responses. The GAD-7 total score was classified into the following groups: Low=0; Moderate=1; High =2.

***Features*.** All potential features (predictors), including associated risk factors and socio-demographic variables were considered in the ML models for the assessment of pregnant women before and during the COVID-19 lockdown. The socio-demographic features included women’s age, age at marriage, country of residence, education level, work status, family income, and locality.

Associated risk factors included pre- and post-COVID-19 pandemic food consumption patterns, smoking status, body mass index (BMI), physical activity, healthy food consumption (fruits, vegetables, meat, grains, and dairy products), unhealthy food consumption (sweets, soft drinks, energy drinks, and fast food), physical activity level, technology-related activities, COVID-19 diagnosis, relatives diagnosed with COVID-19, underlying health conditions (diabetes, gestational diabetes, hypertension, gestational hypertension, heart and arterial diseases, liver diseases, high cholesterol, high triglycerides, thyroid disorders, or respiratory problems), cancellation of follow up appointment due to COVID-19, number of pregnancies, number of abortions, family problems, social problems, psychological stress, and work-related stress.

***Data analysis*.** General descriptive analysis and ANOVA tests were used for describing the distribution of women based on risk factors, while prediction and classifications were measured using ML techniques. The classification accuracy, confusion matrix, precision, sensitivity, and specificity were used for evaluating the ML prediction performance. The ML algorithms were applied in the Python AI development platform to predict the incidence and severity level of depression and anxiety during COVID-19 among pregnant women. The data set was divided into a 70:20:10 ratio for training, testing and validation.

To evaluate whether the ML algorithms can predict pregnant women's depression and anxiety, the outcome variables and features were included in the ML models. The performance of ML models was first evaluated for depression and then for anxiety separately. The performance metrics for Gradient Boosting Machines (GB), Distributed Random Forests (RF), Extreme Randomized Forests (XRT), Naïve Bayes (NB), Support Vector Machine (SVM), Multilayers Neural Network (MNN), and Decision Tree (DT) are presented in the results. The accuracy, precision, Area Under the Curve (AUC), Matthew's Correlation Coefficient (MCC) and Receiver Operating Characteristic Curve (ROC) were used for measuring the performance accuracy.

## Results

Table 2 and Table 3 show the descriptive analysis of the participants’ data by anxiety and depression levels. Results indicated that the women’s mean age was 28.5 (±5.3) years. Among participants, 11.6% and 8.7% had moderate and high levels of depression, respectively while 22.4% and 7.7% had moderate and high levels of anxiety, respectively.

**Table 2.** Descriptive analysis of study variables and maternal depression.

	Category	Level of Depression				
		No Depression	Moderate	High	F	P-Value
		**n(row%)**				
**The country of residence**	Jordan	229(50.0)	133(29.0)	96(21.0)	7.9	<0.001
	Palestine	208(49.3)	119(28.2)	95(22.5)		
	Lebanon	232(59.2)	88(22.4)	72(18.4)		
	Saudi Arabia	120(61.5)	47(24.1)	28(14.4)		
	Bahrain	89(66.4)	26(19.4)	19(14.2)		
**Locality**	Urban	594(54.0)	285(25.9)	221(20.1)	1.1	.285
	Non-Urban	284(56.7)	128(25.5)	89(17.8)		
**Women age (years)**	<35	744(54.7)	350(25.7)	267(19.6)	0.4	.542
	≥35	133(55.6)	63(26.4)	43(18.0)		
**Age at marriage (years)**	<20	142(53.4)	72(27.1)	52(19.5)	1.1	.324
	20–29	671(54.5)	317(25.8)	243(19.7)		
	≥30	64(62.1)	24(23.3)	15(14.6)		
**Education level**	≤Secondary school	175(49.6)	96(27.2)	82(23.2)	2.8	.095
	> Secondary school	703(56.3)	317(25.4)	228(18.3)		
**Currently working**	Yes	357(59.7)	133(22.2)	108(18.1)	2.9	.090
	No	521(51.9)	280(27.9)	202(20.1)		
**Family income**	Decreased	489(50.0)	267(27.3)	222(22.7)	22.7	.000
	Increased/same	389(62.4)	146(23.4)	88(14.1)		
**Physical activity during pandemic**	Inactive (<1/2 hour per day))	323(57.6)	124(22.1)	114(20.3)	0.5	.488
	Active (≥1/2 hour per day)	546(53.6)	279(27.4)	194(19.0)		
**Food group adherence during** ** pandemic**	No/low adherence (0–2)	429(53.5)	223(27.8)	150(18.7)	0.5	.494
	Moderate/high adherence (3–5)	115(55.6)	53(25.6)	39(18.8)		
**Smoking during pandemic**	Non-smoker	495(56.8)	200(22.9)	177(20.3)	10.6	.001
	Smoker	62(48.4)	40(31.3)	26(20.3)		
**Number of pregnancies**	One	305(56.5)	139(25.7)	96(17.8)		
	Two	236(53.9)	117(26.7)	85(19.4)	.509	.676
	Three	158(55.6)	62(21.8)	64(22.5)		
	Four +	178(52.7)	95(28.1)	65(19.2)		
**Number of miscarriages**	Zero	638(54.2)	316(26.8)	223(18.9)		
	One time	162(55.7)	69(23.7)	60(20.6)	.202	.895
	Two Time +	77(58.3)	28(21.2)	27(20.5)		
**Sleeping hours during pandemic**	<6	58(49.6)	22(18.8)	37(31.6)		
	6–8	429(58)	181(24.5)	130(17.6)	5.8	.003
	>8	388(52.6)	207(28.1)	142(19.3)		
**Has been diagnosed with COVID-19**	No	826(55.5)	383(25.7)	279(18.8)		
	Yes	52(46.0)	30(26.5)	31(27.4)	4.5	.034
**Has any of your relatives been** ** diagnosed with COVID-19**	No	720(55.9)	322(25.0)	246(19.1)		
	Yes	158(50.5)	91(29.1)	64(20.4)	0.3	.572
**Chronic disease**	No	791(55.0)	375(26.1)	273(19.0)		
	Yes	87(53.7)	38(23.5)	37(22.8)	0.0	.886
**Stress during pandemic**	No	177(85.5)	19(9.2)	11(5.3)		
	Yes	701(50.3)	394(28.3)	298(21.4)	130.8	<0.001
**Family problems**	No	754(58)	343(26.4)	204(15.7)		
	Yes	124(41.3)	70(23.3)	106(35.3)	78.2	<0.001
**Financial problems**	No	665(59.0)	293(26.0)	169(15.0)		
	Yes	213(44.9)	120(25.3)	141(29.7)	72.7	<0.001
**Social problems**	No	805(54.9)	385(26.3)	275(18.8)		
	Yes	73(53.7)	28(20.6)	35(25.7)	8.0	.005
**Psychological problems**	No	698(60.9)	277(24.2)	171(14.9)		
	Yes	180(39.6)	136(29.9)	139(30.5)	111.1	<0.001
**Work stress**	No	749(55.5)	349(25.9)	252(18.7)		
	Yes	129(51.4)	64(25.5)	58(23.1)	8.1	.004

**Table 3.** Descriptive analysis of study variables and maternal anxiety.

		Level of Anxiety				
	Category	No Anxiety	Moderate	High	F	P-Value
			**n(row%)**			
**Country of residence**	Jordan	208(36.7)	253(44.7)	105(18.6)	4.9	.001
	Palestine	132(31.2)	207(48.9)	84(19.9)		
	Lebanon	154(39.0)	186(47.1)	55(13.9)		
	Saudi Arabia	75(38.5)	97(49.7)	23(11.8)		
	Bahrain	68(50.4)	58(43.0)	9(6.7)		
**Locality**	Urban	407(35.7)	539(47.3)	193(16.9)	4.6	.033
	Non-urban	230(40.0)	262(45.6)	83(14.4)		
**Women age (years)**	<35	535(36.9)	681(46.9)	235(16.2)	0.3	.608
	≥35	102(38.9)	119(45.4)	41(15.6)		
**Marriage age (years)**	<20	113(37.0)	131(43.0)	61(20.0)	2.1	.128
	20–29	476(36.6)	620(47.7)	203(15.6)		
	≥30	48(44.0)	49(45.0)	12(11.0)		
**Education level**	≤Secondary school	115(30.5)	172(45.6)	90(23.9)	21.3	<0.001
	> Secondary school	522(39.0)	629(47.0)	186(13.9)		
**Currently working**	Yes	268(40.7)	314(47.7)	76(11.6)	14.1	<0.001
	No	369(34.9)	487(46.1)	200(18.9)		
**Family income**	Decreased	355(33.0)	519(48.2)	203(18.8)	18.0	<0.001
	Increased/same	282(44.3)	282(44.3)	73(11.5)		
**Physical activity during pandemic**	Inactive (<1/2 hour per day)	225(38.7)	267(46.0)	89(15.3)	1.9	.165
	Active (≥1/2 hour per day)	406(36.5)	526(47.3)	180(16.2)		
**Food group adherence during ** **pandemic**	No/low Adherence (0-2)	325(36.4)	409(45.8)	159(17.8)	0.0	.945
	Moderate/high Adherence (3-5)	79(37.3)	102(48.1)	31(14.6)		
**Smoking during pandemic**	Non-smoker	313(35.7)	425(48.5)	138(15.8)	1.2	.267
	Smoker	38(29.7)	66(51.6)	24(18.8)		
**Number of pregnancies**	One	216(38.6)	259(46.3)	85(15.2)		
	Two	168(36.6)	222(48.4)	69(15.0)	.078	.925
	Three	112(36.7)	141(46.2)	52(17.0)		
	≥Four	141(36.2)	178(45.8)	70(18.0)		
**Number of miscarriages**	Zero	461(36.5)	597(47.3)	204(16.2)		
	One time	116(37.5)	149(48.2)	44(14.2)	.189	.827
	≥Two times	60(42.3)	54(38.0)	28(19.7)		
**Sleeping hours during pandemic**	<6	36(29.0)	56(45.2)	32(25.8)	5.1	.006
	6–8	324(40.2)	361(44.8)	121(15.0)		
	>8	275(35.4)	381(49.0)	121(15.6)		
**Has been diagnosed with COVID-19**	No	597(37.5)	748(46.9)	249(15.6)	2.0	.158
	Yes	40(33.3)	53(44.2)	27(22.5)		
**Has any of your relatives been** ** diagnosed with COVID-19**	No	538(38.7)	633(45.6)	218(15.7)	8.6	.003
	Yes	99(30.5)	168(51.7)	58(17.8)		
**Chronic diseases**	No	558(36.4)	727(47.5)	246(16.1)	1.2	.275
	Yes	79(43.2)	74(40.4)	30(16.4)		
**Stress during pandemic**	No	251(79.4)	55(17.4)	10(3.2)	253.3	<0.001
	Yes	386(27.6)	746(53.4)	265(19.0)		
**Family problems**	No	572(40.5)	665(47.1)	176(12.5)	102.5	<0.001
	Yes	65(21.6)	136(45.2)	100(33.2)		
**Financial problems**	No	537(43.3)	539(43.5)	164(13.2)	72.9	<0.001
	Yes	100(21.1)	262(55.3)	112(23.6)		
**Social problems**	No	597(37.9)	732(46.5)	246(15.6)	7.2	.007
	Yes	40(28.8)	69(49.6)	30(21.6)		
**Psychological problems**	No	543(43.2)	540(43.0)	173(13.8)	76.3	<0.001
	Yes	94(20.5)	261(57.0)	103(22.5)		
**Work stress**	No	578(39.5)	649(44.4)	235(16.1)	13.9	<0.001
	Yes	59(23.4)	152(60.3)	41(16.3)		

The rates of anxiety and depression were found to differ by the country of residence, education level, family income, work stress, social problems, health problems, family problems, financial problems, psychological problems, sleeping hours per day, fear of COVID-19 infection, and unhealthy food consumption. A greater percentage of women with high levels of depression and anxiety symptoms were found in Palestine (19.9%, 22.5%) and Jordan (18.6%, 21.0%), respectively. Furthermore, the results in Table 2 indicated that the highest percentages of depression were among women with self-reported family problems (35.3%), sleep deprivation (<6 hours per night) (31.6%), psychological problems (30.5%), financial problems (29.7%), COVID-19 diagnosis (27.4%), social (25.7%), and work stress (23.1%). Furthermore, high levels of anxiety symptoms were found among women with self-reported family problems (33.2%), financial (23.6%), social (21.6%), and psychological problems (22.5%) as shown in Table 3.

### ML performance measures

Different performance measures were considered in our study to evaluate whether the ML models can predict women’s depression and anxiety symptoms during the COVID-19 lockdown. Seven ML classification algorithms were tested on our dataset, including SVM, K-nearest neighbour (KNN), NB, Random Forest (RF), Neural Network (NN), DT, and GB. The performance was evaluated using several assessments measures such as accuracy, precision, Area Under the Curve (AUC), Matthew's Correlation Coefficient (MCC) and Receiver Operating Characteristic Curve (ROC). The performance measures were calculated using the following equations:

1. Specificity

$$Specificity=\frac{TN}{FP+TN}$$

2. Precision

$$Precision=\frac{TP}{TP+FP}$$

3. Recall

$$Recall=\frac{TP}{TP+FN}$$

4. F-measure

$$FM=\frac{2*precision*recall}{precision+recall}$$

5. Matthew’s Correlation Coefficient

$$MCC=\frac{\left(TN*TP)-(FN*FP\right)}{\left(FP+TP)(FN+TP)(TN+FN\right)}$$

6. Accuracy

$$Accuracy=\frac{TP+TN}{TP+TN+FP+FN}$$

**Figure 1.**
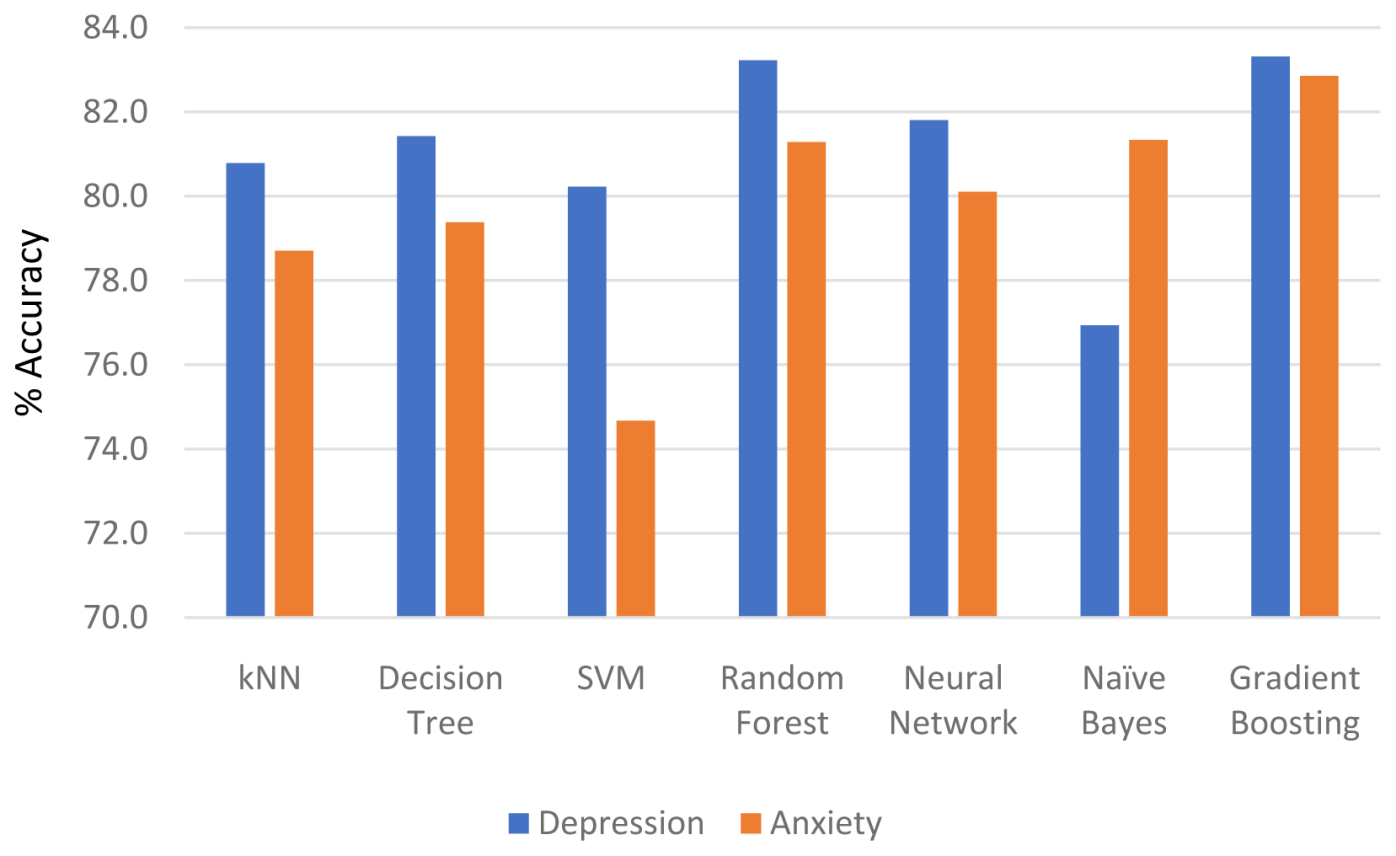
Percentage of ML models accuracy by depression and anxiety symptoms.

Figure 1 represents the comparison of accuracy rates among the selected machine learning algorithms in predicting women's depression and anxiety symptoms. All tested models reported a high level of accuracy (ranging from 80.0–83.3%) for predicting depression among pregnant women except for NB. On the other hand, various levels of accuracy were reported for the ML models when predicting anxiety. The GB model presented the highest accuracy rate (82.9%) followed by RF and NB (81.3%). Nonetheless, all the ML models reported an acceptable rate of accuracy for both depression and anxiety symptoms.

**Figure 2.**
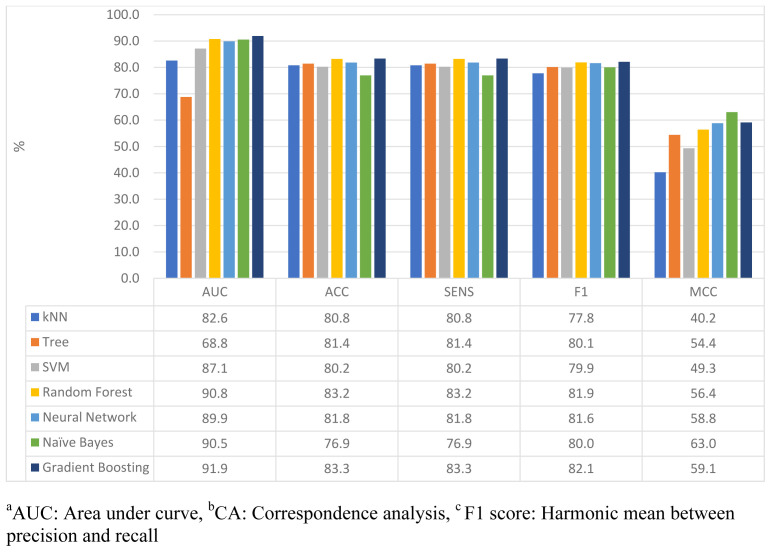
Evaluation of ML models performance analysis in predicting depression symptoms.

Additional performance measures were used for evaluating the ML prediction performance of depression and anxiety symptoms, including AUC, sensitivity, specificity, F-Measure, and MCC. Figure 2 illustrates the different performance measures of depression prediction models. Balanced accuracy, sensitivity, and F measures were observed across the ML models. The AUC varied across models; DT reported the lowest AUC rate (68.8%), while other models ranged from 82.6% to 91.9%. The MCC performance measure showed high variability across models being relatively low among the different ML models; the NB model reported the highest MCC value of 63%. Overall, GB reported the highest AUC, ACC, sensitivity, and F1 measures among all other ML models.

**Figure 3.**
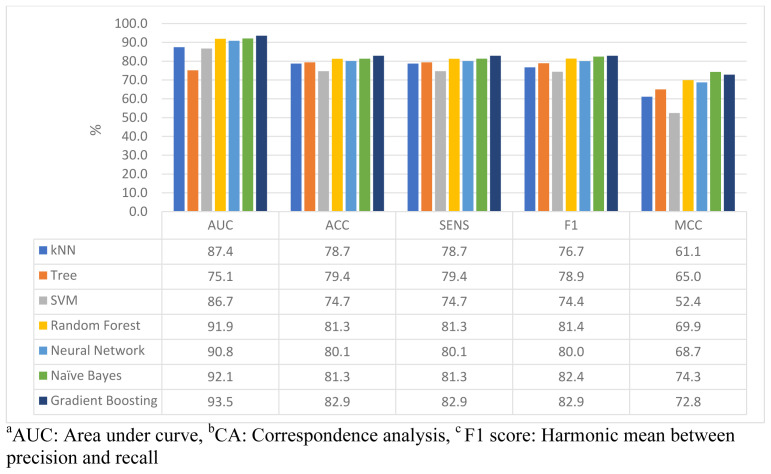
Evaluation of ML models performance analysis in predicting anxiety symptoms.

Figure 3 shows the different performance measures of ML models for the anxiety prediction. Performance analysis for the anxiety prediction reported quite similar AUC, ACC, sensitivity, and F1 measures for the RF, NN, KNN, and GB models. SVM and DT models had the lowest accuracy measures. The sensitivity and accuracy were highest at the GB and NB models. The MCC performance measure varied among the ML models, the highest of which were found in the NB and GB models (74.3%, 72.8%, respectively). The SVM had the lowest MCC performance measure (52.4%). Overall, GB achieved the best accuracy and sensitivity, and F1-measures of 82.9%, and a balanced MCC measure of 72.8%.

**Figure 4.**
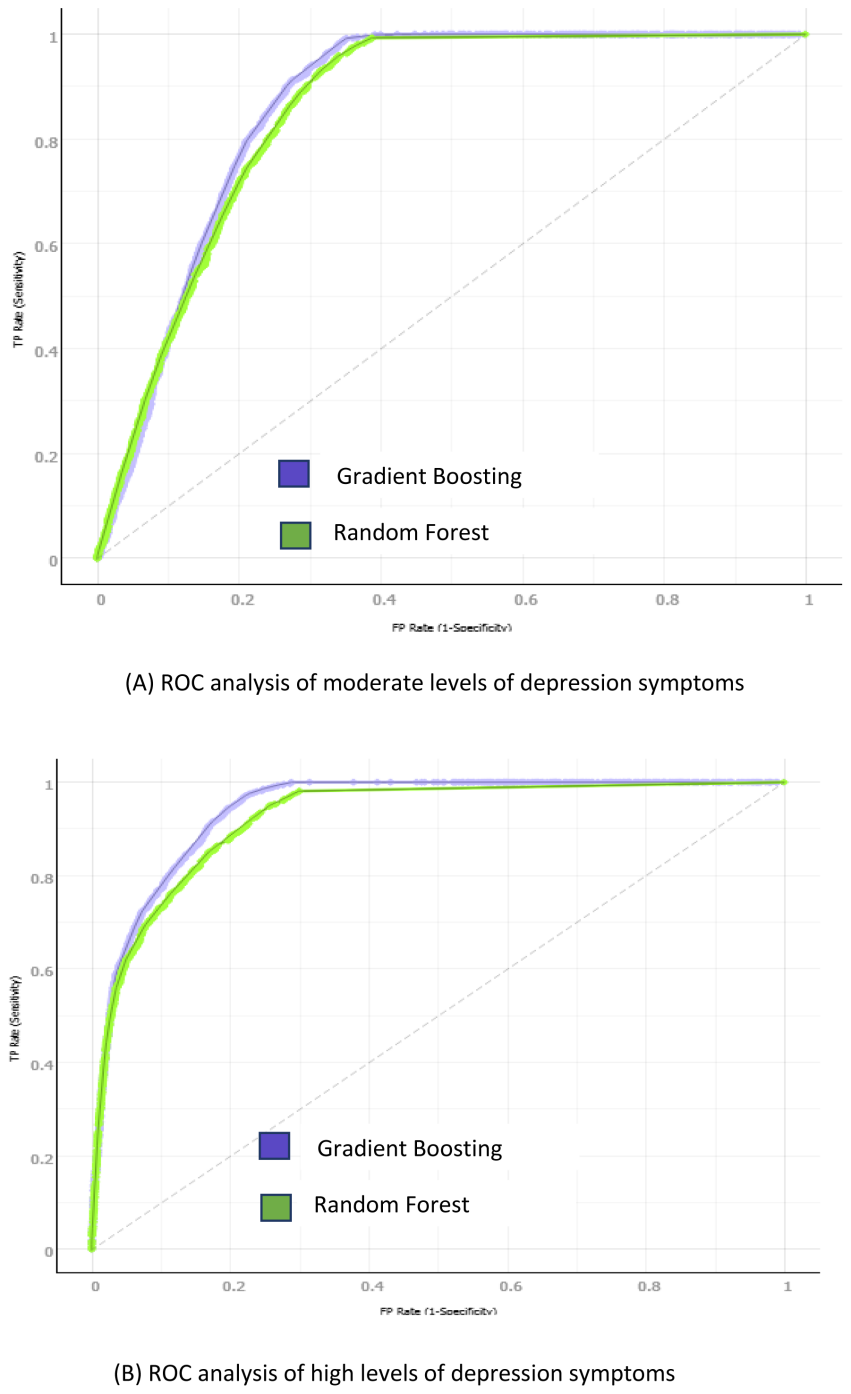
Gradient Boosting and Random Forest ROC sensitivity and specificity analysis: ( **A**) Moderate depression symptoms analysis; ( **B**) High depression symptoms analysis.

**Figure 5.**
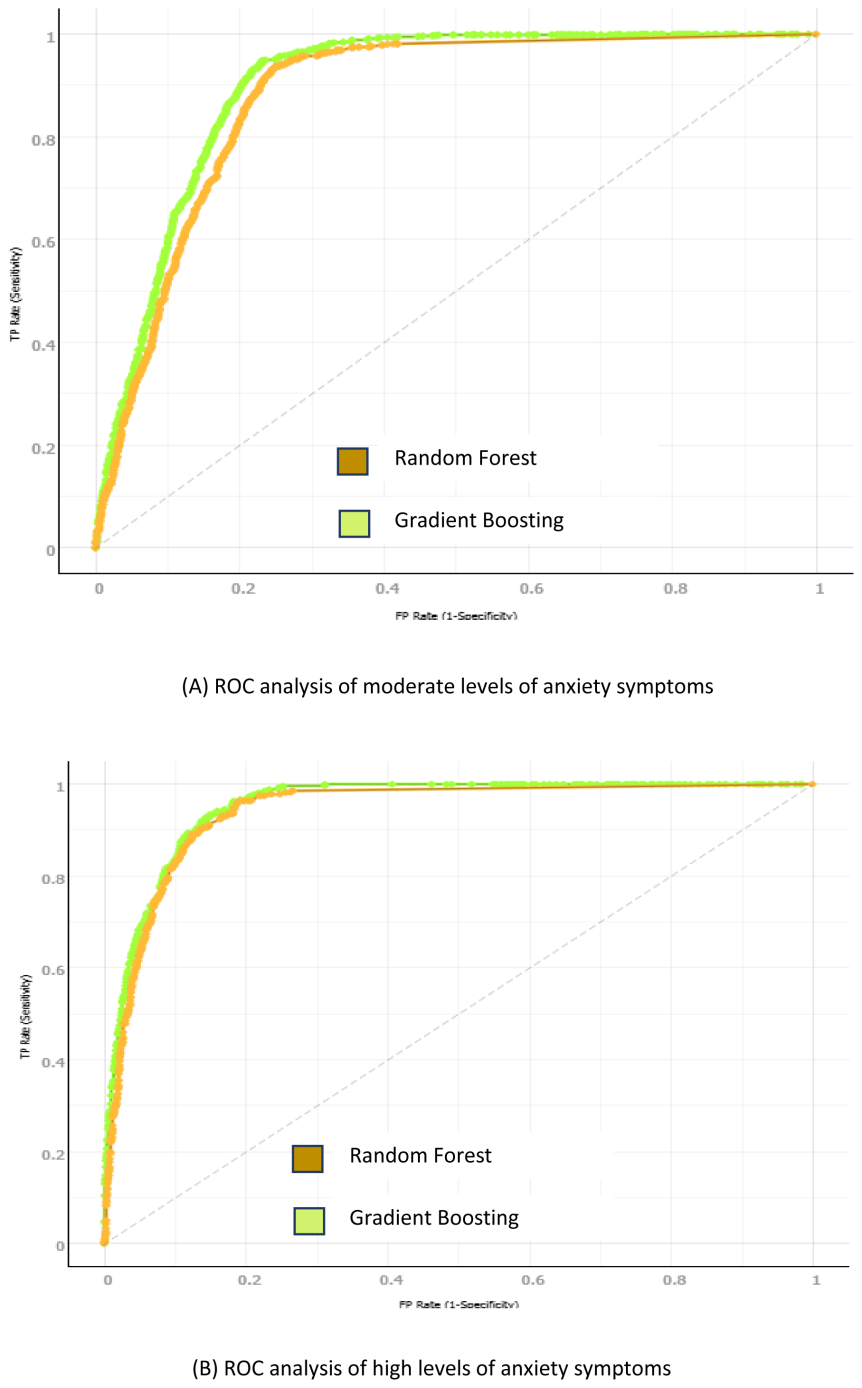
Gradient Boosting and Random Forest ROC sensitivity and specificity analysis: ( **A**) Moderate anxiety symptoms analysis; ( **B**) High anxiety symptoms analysis.

The GB and RF receiver operating characteristics (ROC) for the moderate and high depression and anxiety classes is presented in Figure 4 (A and B) and Figure 5 (A and B) respectively. Three numerical categories of student depression and anxiety classes were used: low, moderate, and high. The ROC resides in the upper left corner; thus, the gradient boosting algorithm showed a better prediction of positive value than the other studied algorithms (AUC of 91.9% and 93.5% for depression and anxiety, respectively).

### Features’ importance

**Figure 6.**
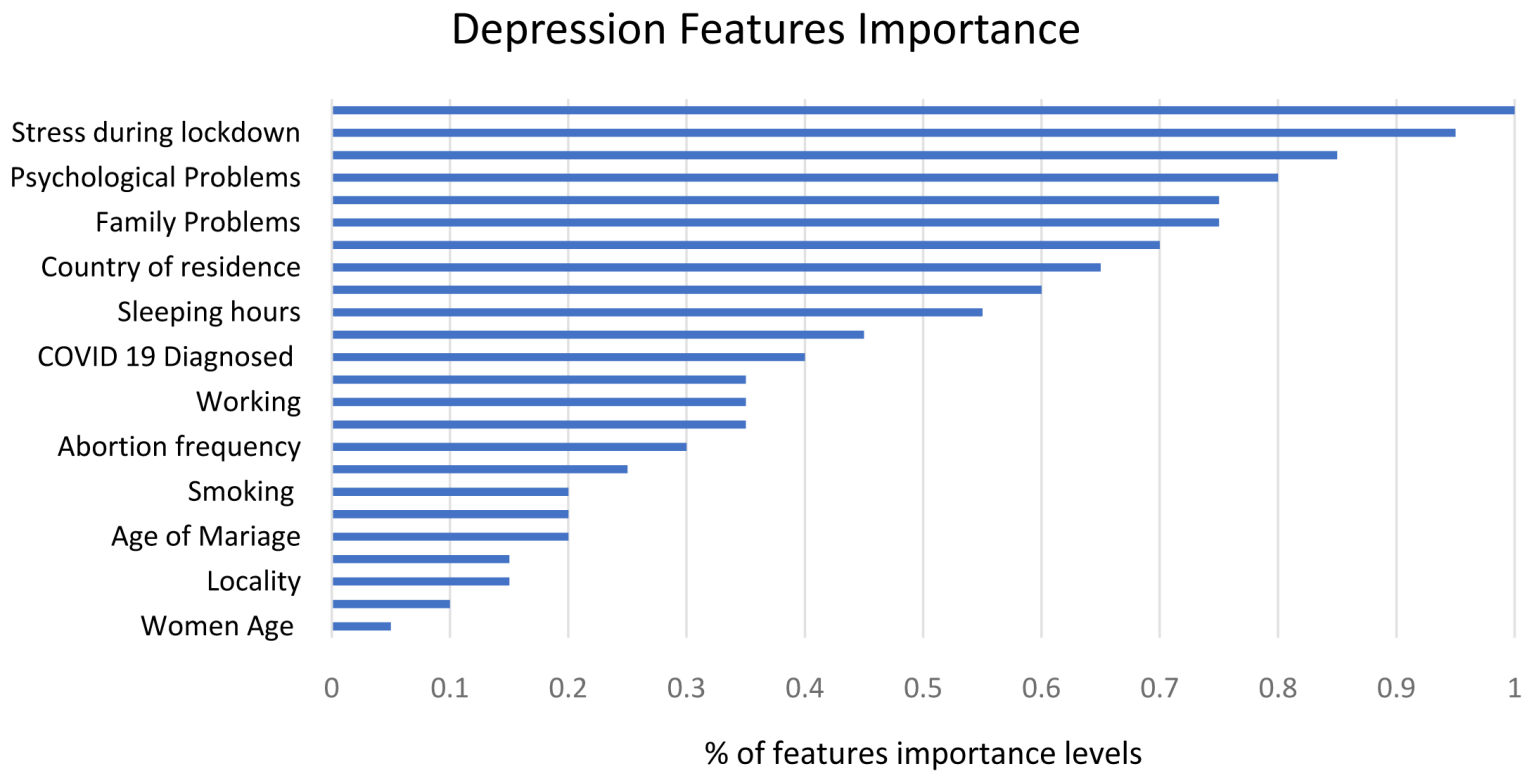
Variables' importance ranking analysis in predicting depression symptoms.

**Figure 7.**
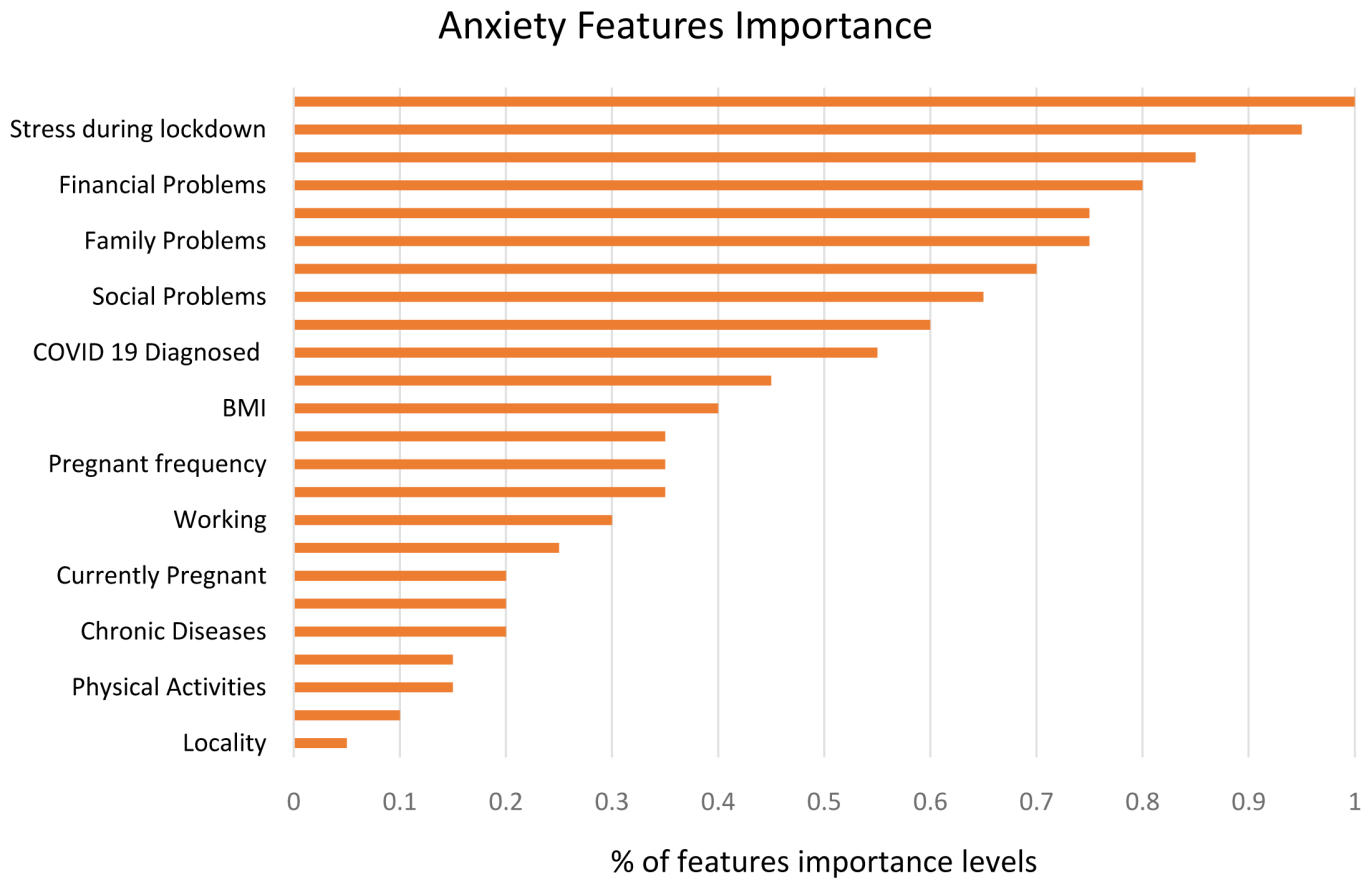
Variables' importance ranking analysis in predicting anxiety symptoms.

The 23 variables used for predicting depression and anxiety symptoms in the ML models were classified and ranked from 0 to 100%. The variables with importance level greater than 60% were considered. The participants reported different levels of variables’ importance for depression and anxiety. The distribution of most important variables for depression and anxiety can be found in Figure 6 and Figure 7, respectively. The most significant variables in predicting depression symptoms were stress during lockdown, psychological factors, family problems, and country of residence. While the most significant variables in predicting anxiety were stress during lockdown, financial problems, family problems, social problems, and COVID-19 diagnosis.

## Discussion

In this study, we used machine learning techniques for the prediction of depression and anxiety among pregnant and postpartum women from five Middle Eastern countries (Lebanon, Palestine, Jordan, Bahrain, and Saudi Arabia) during the COVID-19 lockdown. We found that 20.3% of women had moderate to severe maternal depression while 30.1% of them had moderate to severe anxiety, the highest rates being among Palestinian and Jordanian women. The findings of this study are consistent with other studies that indicated high levels of anxiety and depression symptoms among pregnant women during COVID-19 lockdown ^3^. Women reported a significant concern and were found at high risk of developing post-traumatic stress disorder, which requires direct intervention from health care providers for caring of pregnant and postpartum women mental health during COVID-19 pandemic.

The performance of different ML models in predicting maternal depression and anxiety was evaluated through measuring accuracy, specificity, precision, recall, F-measure, and Matthew's Correlation Coefficient (MCC). The accuracy performance of the studied models was similar and did not indicate a significant difference across models. GB and RF reported the best accuracy, sensitivity, and F1 measures for depression prediction. The MCC has been measured for the selected models as an alternative performance measure which is not affected by an unbalanced dataset. The MCC measure showed acceptable scores for both depression and anxiety symptoms. The NB had the highest MCC values followed by GB and RF. Thus, the results in this study are consistent with other studies that assessed the performance of ML classifiers in predicting depression among pregnant and postpartum women, where the RF model showed the highest accuracy and AUC values ^16, 24^.

The ML prediction models of postpartum depression developed by Shin *et al*., (2020) achieved an AUC of 0.79. On the other hand ^29^, utilized a multilayer perceptron approach using several risk factors for depression prediction among Spanish pregnant and postpartum women. The model accomplished an AUC value of 0.82, sensitivity value of 0.84, and specificity of 0.81. Furthermore, in a study, Logistic Regression (LR) classifier was used for depression prediction and achieved an accuracy value of 83.3% ^30^ while employing multiple ML algorithms including KNN, LR, Linear Discriminant Analysis (LDA), and B improved the overall accuracy values to 90% ^28, 29^. Additionally, our study was consistent with 24 study in which the ML classifiers were used in predicting postpartum depression and found that depression before pregnancy, stress during pregnancy, and smoking were the most significant risk factors for depression. On the contrary of our findings ^31^, reported that women’s age, marital status, and education were the most significant factors relating to postpartum depression. 

Furthermore, our study reported a higher AUC performance measure than other similar ML prediction studies, whose AUC measures were 80% ^32^, 79% ^5^, and 78% ^33^. The results in our study showed an accuracy of 83.3%, which is comparable to the 84% accuracy rate reported in other studies ^26, 30^. Nevertheless, the study sample used in this research was collected from diverse population groups across countries, thus diverging background and environmental factors were expected to affect the homogeneity of the dataset.

Significant risk factors for pregnant and postpartum depression and anxiety were found, including country of residence, family income, smoking, COVID-19 diagnosis, number of hours of sleep, stress during the COVID-19 lockdown, family support, social support, financial situation, psychological problems, and work stress. Additionally, risk factors particularly significant for anxiety included education level, locality, and work status. We found the rates of anxiety symptoms to be higher than those of depression among pregnant and postpartum women during the pandemic lockdown. The results showed that Jordanian, Palestinian and Lebanese women had higher anxiety and depression than Saudi and Bahraini women. The increased risk for depression and anxiety among women could be explained by low family income, financial problems, and poor healthcare systems available in these countries ^34^. The study also reported significant differences in anxiety because of locality and education levels. Women with lower education levels reported higher anxiety; similarly, women living in urban areas presented higher anxiety levels. These findings could be explained by the stricter lockdown in cities and the lack of knowledge about the disease among women with lower education levels. 

The machine learning models returned the five highest ranking features affecting women’s depression symptoms: stress during pregnancy, psychological problems, family support, country of residence, and number of hours of sleep-in descending order. The highest-ranking features for anxiety were stress during pregnancy, financial problems, family problems, social problems, and COVID-19 diagnosis. Our findings are consistent with similar studies indicating that stress during pregnancy negatively affects women's mental health and might influence incidence of postpartum depression ^9, 14, 32^. Furthermore, the results were consistent with other studies indicating that family income, and social and psychological problems had significant impact on maternal mental health ^3, 6, 8^.

The study provides an interesting finding that the accuracy performance measures is relatively high and remains stable between the selected ML models, especially for AUC, accuracy, and sensitivity even at reduced number of variables. This finding is consistent with other studies ^17, 31, 34– 36^ that indicated the high correlation between anxiety and depression symptoms and other socio-demographic risk factors. Thus, stress, family support, financial situation, psychological problems, and country of residence were among the most important variables associated with depression and anxiety during the pandemic lockdown. This is important to consider when developing intervention strategies and programs. The stability in performance measures reflects that the self-reported survey methods can be used as a good assessment tool for anxiety and depression. Moreover, pregnant women had more anxiety symptoms than depression during lockdown, which might affect maternal and child health.

Our findings suggest that deploying machine learning techniques for the screening of pregnant and postpartum women will help in identifying those at highest risk of anxiety and depression through clustering and classification, which will in turn aid in the development of effective preventive interventions. Thus, this research not only addresses the integration of innovative technology for the prediction and diagnosis of depression and anxiety among pregnant and postpartum women in low- and middle-income countries, but given the international dataset used, it assesses the prediction power of several ML algorithms across diverging population groups with distinct risk factors. Additionally, the study included variables specific to the COVID-19 lockdown period, which differentiates it from similar studies.

Nevertheless, some limitations are found in this study, including the extent of the study sample. Having a smaller dataset limits the power of predictions to train a robust range of algorithms, as well as limits the number of clusters and classifications produced by the ML predictive models. In addition, the study used the online self-reported assessment, which was not fully completed by all study participants. Nonetheless, the incomplete and missing data were excluded from out dataset. Finally, a more comprehensive study with a larger and more representative dataset including clinical data is recommended for future research among low- and middle-income countries.

## Conclusion

The study assessed the performance measures of machine learning algorithms in predicting depression and anxiety among pregnant and postpartum women in low- and middle-income countries during the COVID-19 pandemic lockdown. Based on the results presented, this research concludes that ML algorithms, particularly (yet not exclusively) Gradient Boosting and Random Forest, are effective predictive models for maternal mental health. These models could be integrated into clinical medical information systems for the automatic prediction of pregnant women’s depression and anxiety based on the identified key variables. The deployment of ML models will provide effective clinical applications for the development of prevention and intervention programs. Likewise, by making use of accurate machine learning techniques such as Random Forest, public health professionals, healthcare providers, and decision-makers will be able to predict rising issues and implement relevant intervention programs to enhance maternal and child health in their respective countries.

## Data availability

### Underlying data

Harvard Dataverse: Pregnancy and Mental Health Data during COVID-19

https://doi.org/10.7910/DVN/FCDGEB ^27^

This project contains the following underlying data:

- ML-DataSet.xlsx
- Variables_Descriptions.xlsx

### Extended data

Dataverse: Pregnancy and Mental Health Data during COVID-19

https://doi.org/10.7910/DVN/FCDGEB ^27^

This project contains the following extended data:

- English questionnaire.xlsx

Data are available under the terms of the Creative Commons Zero "No rights reserved" data waiver (CC0 1.0 Public domain dedication).
