# Supplementary material for: Machine learning techniques for predicting depression and anxiety in pregnant and postpartum women during the COVID-19 pandemic: a cross-sectional regional study
Source: F1000Res. 2022 Apr 4;11:390. [Version 1] doi: 10.12688/f1000research.110090.1 (PMC9445566; doi:10.12688/f1000research.110090.1)
Supplement: Peer review history

# Peer review: Reviewer response for version 1

**DOI:** 10.5256/f1000research.121665.r144638

**Published:** 2022-09-05

**Authors:** Iyad Tumar

**Affiliations:** 1 Birzeit University, Birzeit, Palestinian Territory

The study used the machine learning techniques in predicting the effect of COVID 19 on the women depression and anxiety. The machine learning models used original data set collected from Arab countries during COVID19-pandemic lockdown. The study sample composed of 3569 women (1939 pregnant and 1630 postpartum).  The study indicated that the gradient boosting algorithm reported the highest performance compared to other algorithms.

 The study addressed an important problem in developing countries, and the result of the study is very encouraging, mainly in the deployment of machine learning in the fields of Mental health and public health.   Furthermore, the study is well-written and organized. However, a few issues need more clarifications:

1. The study contains many figures and tables, will be much better to add some of them in the Appendix.
2. I recommend double checking the samples and features of Table 2.
3. Are there any significant differences between pregnant women and postpartum?
4. Does the authors use a weighted dataset to analyze and run the ML models.
5. It is important to show the overfitting problem during the training and testing.

Is the work clearly and accurately presented and does it cite the current literature?

Yes

If applicable, is the statistical analysis and its interpretation appropriate?

Yes

Are all the source data underlying the results available to ensure full reproducibility?

Yes

Is the study design appropriate and is the work technically sound?

Yes

Are the conclusions drawn adequately supported by the results?

Yes

Are sufficient details of methods and analysis provided to allow replication by others?

Yes

Reviewer Expertise:

My research area is in the fields of Artificial Intelligence (AI). Within AI, I am interested in problems related to health or education modeling, machine learning, and data mining, and their interdisciplinary applications to real-life problems. Furthermore, I’m interested in computer networks and cybersecurity, in which I worked on the development of models and methods of network management and protection.

I confirm that I have read this submission and believe that I have an appropriate level of expertise to confirm that it is of an acceptable scientific standard.

# Peer review: Reviewer response for version 1

**DOI:** 10.5256/f1000research.121665.r136365

**Published:** 2022-06-08

**Authors:** Chadi Ibrahim Fakih

**Affiliations:** 1 IVF Department, Al-Hadi Laboratory and Medical Center, Beirut, Lebanon

The context of the work proposed in this article is to use machine learning techniques to predict maternal depression and anxiety levels during the COVID-19 pandemic. The data are collected from five Arab countries and composed of 3569 samples (1939 pregnant women and 1630 postpartum). Several machine learning techniques are applied such as Random Forest, Gradient Boosting and Naïve Bayes. As a result, the gradient boosting algorithm outperformed the other methods used in this research.

 Regarding the form, the article is well organized and written. The addressed problem is quite important and the results obtained are promising. However, the article suffers from several weak points:

1. How are features’ importance evaluated? (filtering method) Do authors focus on filter-based, wrapper-based or embedded-based methods? It seems they have based on embedded one, but they should be precise.
2. Why feature extraction methods are not used such as LDA, ICA and t-SNE?
3. Article contains a lot of figures and tables per evaluation measure. I think these figures and tables can be put in the appendix and focus on one evaluation measure such as accuracy.
4. You may provide a matrix that shows the correlation between each couple of features and between each feature and the different classes.
5. What is the ^b^CA mentioned under the figures 2 and 3?  (Component analysis or classification accuracy).
6. Authors should show a comparison of their work with other works even they are not applied on the same dataset. It is important to see how the efficiency of each machine learning varies between the work and other works.
7. Authors stated that they used 3569 samples in their study while when we refer to the table 2 and table 3 there is something missing. For example, if we count in table 2 all the samples in the row that corresponds to the country of residence regardless the depression level, they sum to 1601. In contrast, the sum of the samples in the row smoking during pandemic is 1000 and others sums to 1600. Are there missing features’ values?
8. There is something missing in table 2 (table 3 also). Table 2 shows that the total samples belonging to class “No depression” is 878 over 1601, “Moderate depression” is 413/1601 and “High depression” is 310/1601. Is the total number of samples are 1601? I thought they are 3569.
9. In page 5 – section “Results”, you mention “Among participants, 11.6% and 8.7% had moderate and high levels of depression, respectively while 22.4% and 7.7% had moderate and high levels of anxiety, respectively”. It means, the class “No depression” constitute the 80% of the dataset and “No anxiety” 70% of the dataset. How is this class-imbalance handled during experimentations?
10. Put the confusion matrix for depression-prediction and the one for anxiety-prediction to see how the misclassified samples are distributed.
11. The dataset is split into 70:20:10 (training:testing:validation). What type of parameters did you use that need validation? Did you split them randomly into three groups? You should use techniques other than random sampling such as cross-validation.
12. Authors should show the training error to compare it with the testing error in order to show if there is overfitting.
13. No need to put the formulas of the evaluations measures (Precision, recall, AUC,…). They are known.
14. Gradient-boosting and Random-forest are decision-tree based classifiers. It is important to derive the rules that constitute the model showing the set of rules of each class.

Is the work clearly and accurately presented and does it cite the current literature?

Yes

If applicable, is the statistical analysis and its interpretation appropriate?

Yes

Are all the source data underlying the results available to ensure full reproducibility?

Yes

Is the study design appropriate and is the work technically sound?

Yes

Are the conclusions drawn adequately supported by the results?

Yes

Are sufficient details of methods and analysis provided to allow replication by others?

Yes

Reviewer Expertise:

Human reproduction, endocrinology, ART

I confirm that I have read this submission and believe that I have an appropriate level of expertise to confirm that it is of an acceptable scientific standard.
